# Effect of contamination of bulk-fill flowable resin composite with different contaminants during packing on its surface microhardness and compressive strength: in vitro study

**DOI:** 10.1186/s12903-022-02495-6

**Published:** 2022-10-17

**Authors:** Nawal Hassan Aidaros, Ahmed Abdou

**Affiliations:** 1 Restorative Dentistry Department, Faculty of Dentistry, Ahram Canadian University (ACU), Industrial Zone, 6th of October City, Giza, Egypt; 2 Division of Biomaterials, Prosthetic Dentistry Department, Faculty of Dentistry, King Salman International University, El Tur, South Sinai Egypt

**Keywords:** Alcohol, Bulk-fill resin composite, Compressive strength, Contamination, Gloves, Hemostatic agent, Microhardness, Saliva

## Abstract

**Background:**

Proper isolation and restoration of class V subgingival cavities are technique sensitive, thus the resin composite restoration is liable to contamination. This in vitro study was conducted to evaluate the surface microhardness and compressive strength of bulk-fill flowable resin composite after being contaminated during its packing.

**Methods:**

Resin composite discs were prepared using split mold. The contaminated specimens were allocated into four groups (n = 20) according to the contaminant used: hemostatic agent (Group 1), alcohol (Group 2), artificial saliva (Group 3) and powdered gloves (Group 4). The non-contaminated specimens (n = 20) were used as control group. The surface microhardness and compressive strength of each group were tested 1-day post-photocuring (n = 5) and 1 month post-photocuring (n = 5). Values were presented as mean, standard deviation values and confidence intervals.

**Results:**

The surface microhardness of all groups didn’t show a significant difference for different tested groups except for alcohol which showed a significant reduction on surface microhardness compared to control at 1 day post-photocuring (*p* = 0.001). The highest compressive strength mean values at 1 day and 1 month post-photocuring were recorded in control groups (110.42 MPa and 172.87 MPa respectively), followed by alcohol groups, then hemostatic agent groups, followed by artificial saliva with the least value recorded in powdered gloves groups (56.71 MPa and 49.5 MPa respectively).

**Conclusions:**

Contamination of bulk-fill flowable resin composite with hemostatic agent, alcohol, artificial saliva, or powdered gloves during its packing decreased its compressive strength after 1 month post-photocuring rather than affecting its surface microhardness.

**Supplementary Information:**

The online version contains supplementary material available at 10.1186/s12903-022-02495-6.

## Background

Assessing the mechanical properties of resin composite is essential to evaluate the ability of material to survive all challenges present in the oral environment. One of the most useful properties to assess is the surface microhardness because it is correlated with resistance to abrasion [1]. Additionally, the compressive properties of resin-based composites are of great importance as the stress due to mastication is mainly of compressive nature. When parafunctions are present, the compressive stresses are multiplied, leading to fracture of the tooth and restoration [2]. The laboratory testing of compressive strength observes the in vitro fractures that may occur clinically [3]. Flowable resin composite have been preferred by most dentists because of its lower viscosity and subsequent higher flow that allow easier filling the cavity, better adaptation to cavity walls and greater elasticity when compared with other available products [4, 5]. Compared to traditional incremental filling techniques, cavities with a depth higher than 4 mm can be filled through the bulk-fill technique, thus reducing the chair time to fill a cavity [6]. Flowable resin composite has lower modulus of elasticity, thus they are recommended to restore Class V lesions to absorb the mechanical forces during function [7].

Contamination of bulk-fill resin composite during packing could happen accidently when complete proper isolation is difficult to obtain, especially when used to restore class II or class V subgingival caries. Salivary contamination of the cavity can have adverse effects on the longevity of the restoration and may lead to microleakage, sensitivity, tooth discoloration and finally, loss of the restoration [8]. Many studies investigated the effect of contamination of enamel, dentin or adhesive joint with either gloves or saliva on the bond strength of resin composite to tooth structure [9]. Other studies evaluated the effect of contamination of the composite on the incremental layer bond strength during the incremental layering technique [10–12]. However, the current study focused on the contamination effect of resin composite during bulk-filling technique on its mechanical properties. As dentists may rub the hemostatic agent against the tissues before retraction cord application, contamination of the prepared cavity to be restored or the resin composite restoration itself could accidently happen. Many studies investigated the bond strength of enamel and dentin contaminated with hemostatic agent to resin composite [13–15]. However, no studies assessed the effect of contamination of resin composite restoration itself with hemostatic agent during its packing on its mechanical properties. Many clinicians manipulate the resin composite during its packing using either powdered gloves or instruments contaminated with alcohol. Therefore, the aim of this in vitro study was to investigate the effects of contamination of bulk-fill flowable resin composite during packing with different contaminants (Hemostatic agent, artificial saliva, ethyl alcohol and powdered gloves) on its surface microhardness and compressive strength.

## Methods

### Materials

The materials used in this study are shown in Table 1.

**Table 1:** Materials used in the study

	Manufacturer	Composition
Bulk-fill Flowable resin composite	Nexcomp Flow, Meta Biomed, Korea	Nanohybrid flowable composite 40 nanosized filler: Barium Aluminum borosilicate, Bis-GMA, UDMA, Bis-EMA, TMPTMA (75 weight %, 37 volume %)
Hemostatic agent	Hemo Stop solution, JK Dental Vision Company, Egypt	25% Aluminium chloride solution
Artificial saliva		Prepared by mixing of 0.4 g sodium chloride (NaCl), 1.21 g potassium chloride (KCl), 0.78 g sodium dihydrogen dehydrate (NaH_2_PO_4_.2H_2_O), 0.005 g hydrated sodium sulfide (Na_2_S.9H_2_O), and 1 g urea CO(NH_2_)_2_ in 1000 ml deionized water. The pH of this mixture was modified with 10 N sodium hydroxide until it reached 6.7 on a pH meter [15]
Alcohol	Safwa Company, Egypt	70% Ethyl alcohol
Powdered gloves	SRI Trang Argo-Industry Public Company, LTD, Thailand	Powdered Latex examination gloves

### Methods

Experimental in vitro study design was used to investigate the effects of contamination of bulk-fill flowable resin composite during packing with different contaminants.

#### Specimen grouping

A total of 100 specimen of disc-shaped resin composite were prepared using Teflon split mold. The contaminated specimens were allocated into four groups (n = 20) according to the contaminant type used. The non-contaminated specimens (n = 20) were used as control group. Each group was further subdivided into two subgroups of ten specimens each to test the microhardness (n = 10) and the compressive strength (n = 10) at 1-day post-photocuring (n = 5) and 1 month post-photocuring (n = 5).

#### Specimen preparation

Control Group (Non-contaminated group): 20 specimens were prepared by injecting the bulk-fill flowable resin composite into the central hole of Teflon split mold (4 mm diameter × 6 mm height) placed on a clean dry glass slab followed by adequate packing.

The top surface of the resin composite was covered with a celluloid matrix strip (0.05 mm thick); the excess material was removed by pressing a glass slab against the strip [16]. Finally, the resin composite specimen (6 mm height) was photo-cured for 40 s (sec) from the top surface using light emitting diode (LED) light-curing unit of 470 wavelength (Elipar S10, 3 M, ESPE) with light intensity of 1200 mW/cm^2^. The curing protocol performed was standard irradiation at a continuous light-intensity for 40 s, with the light curing tip positioned directly onto the celluloid strip at zero distance.

Group 1–4 (contaminated groups): 20 specimens of each group were prepared by injecting half the specimen height (3 mm) in the lower half of the mold followed by adequate packing. The top surface of the lower half resin composite was contaminated with hemostatic agent (Group 1), alcohol (Group 2), artificial saliva (Group 3) and powdered gloves (Group 4). Groups 1–3; one drop of each contaminant agent was applied on the resin composite surface for 10 s using a clean microbrush in a circular motion. Group 4; powdered gloves were cut into 20 pieces of 1 × 1 cm each [10]. Each piece was applied on the resin composite surface in a circular rubbing motion for 10 s. Then, the upper half of resin composite (3 mm) was injected in the mold. No light curing was performed between the 2 coats. The top of the resin composite was covered with a celluloid strip, and the excess material was removed by pressing the glass slab against the strip. Finally, the resin composite specimen (6 mm height) was photo-cured for 40 s with the light tip positioned directly onto the celluloid strip at zero distance. After removing the specimens from the molds, their dimensions were confirmed using a digital caliper (Mitutoyo MTI Corporation, Tokyo, Japan). The samples were incubated at 37 °C and 95% relative humidity for 1 day and 30 days.

#### Testing the surface microhardness

The microhardness of each group was tested 1-day post-photocuring (n = 5) and 1 month post-photocuring (n = 5). The specimens were tested using Digital Display Vickers Microhardness Tester (Model HVS-50, Laizhou Huayin Testing Instrument Co., Ltd. China) with a Vickers diamond indenter and a 20X objective lens. A load of 100 g was applied on the surface of each specimen for 20 s. Three indentations were made on the surface of each specimen. The indentations were equally placed over a circle, not closer than 0.5 mm to the adjacent indentations. The diagonals length of the indentations was measured by built in scaled microscope and Vickers values were converted into microhardness values. Microhardness was calculated using the following equation: *HV = *1.854* P/d*^*2*^ where, *HV* is Vickers hardness in Kgf/mm^2^, *P* is the load in Kgf and *d* is the length of the diagonals in mm.

#### Testing the compressive strength

The compressive strength of each group was tested 1-day post-photocuring (n = 5) and 1 month post-photocuring (n = 5) using Bluehill® Lite, Instron Instruments. All samples were individually and vertically mounted on a computer-controlled materials testing machine (Model 3345; Instron Instruments Ltd., USA) with a loadcell of 5 kN and data were recorded using computer software (Bluehill Lite; Instron Instruments). Then the samples were statically loaded (in compression manner) using stainless-steel rod ended with flat plate (40 × 60 mm) attached to the upper movable compartment of the machine at a crosshead speed of 0.5 mm/min until failure. The maximum failure load was recorded in N and converted into MPa. The compressive strength was calculated from the recorded peak load divided by sample surface according to the following equation: Compressive strength (CS) = 4P/πd^2^, where P is the load (N) at the fracture point and d is the diameter (mm) of the cylindrical specimen.

#### Statistical analysis

Values were presented as mean, standard deviation (SD) values and confidence intervals. The results of Kolmogorov–Smirnov and Shapiro–Wilk tests indicated that data were normally distributed, therefore Repeated measures analysis of variance (ANOVA) was performed to evaluate the effect of one between-subject variable (groups-contaminants) and one within-subject variable (1 day vs. 1 month). Multiple comparisons with Tukey’s post hoc test was used for comparison between groups (α = 0.05). Statistical analysis was performed with SPSS (IBM SPSS statistics for Windows, v26, IBM Corp). The original data can be accessed in Additional file 1.

## Results

### Surface microhardness test results

**Fig. 1:**
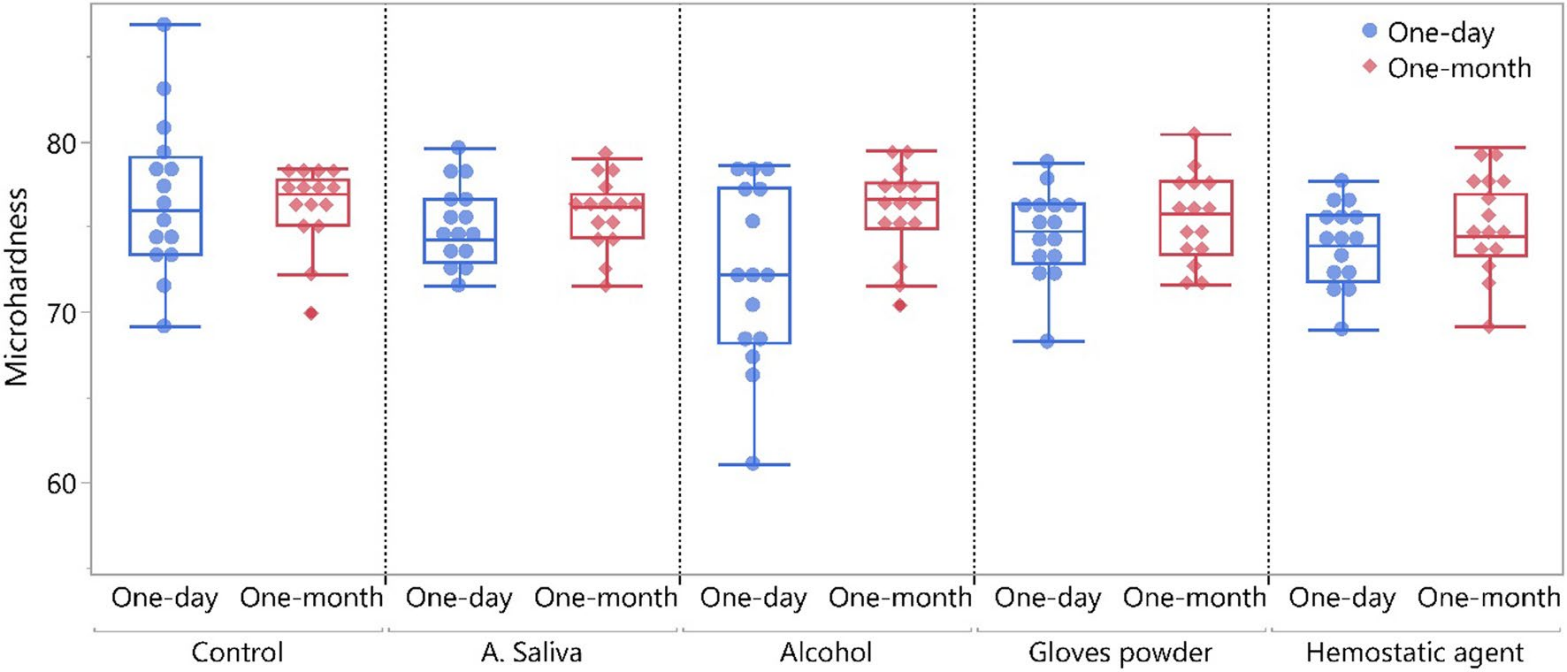
Boxplot showing surface microhardness (HV) values for different tested groups at 1 day and 1 month post-photocuring

The repeated measures ANOVA revealed that storge time had a significant effect on the surface microhardness (*p* = 0.023) while different groups tested and the interaction between groups and storage time resulted in an insignificant effect on the surface microhardness (*p* = 0.052 and 0.118, respectively). The surface microhardness results of tested groups are presented in Table 2 and Fig. 1. At 1-day post-photocuring, control group showed the highest significant surface microhardness value compared to alcohol (*p* = 0.001). While for all other groups, insignificant difference between each other’s resulted (*p* > 0.05). At 1 month post-photocuring, insignificant difference between tested groups resulted at *p* = 0.718. For all groups, insignificant change in surface microhardness resulted after 1 month post-photocuring (*p* > 0.05) except with alcohol group which showed a significant increase in the surface microhardness (*p* = 0.002).

**Table 2:** Microhardness (HV) for tested group at 1 day and at 1 month post-photocuring

	One-day			One-month			*p*-value
	n	Mean ± SD	95%CI	n	Mean ± SD	95%CI	
Control	15	76.73^a^ ± 4.57	74.85–78.62	15	76.16^a^ ± 2.35	74.87–77.45	0.601
A. Saliva	15	75.00^ab^ ± 2.45	73.11–76.88	15	75.78^a^ ± 2.13	74.49–77.07	0.478
Alcohol	15	72.23^b^ ± 5.26	70.34–74.11	15	75.81^a^ ± 2.61	74.52–77.10	0.002
Gloves powder	15	74.58^ab^ ± 2.62	72.69–76.46	15	75.44^a^ ± 2.61	74.15–76.73	0.432
Hemostatic agent	15	73.90^ab^ ± 2.39	72.01–75.79	15	74.92^a^ ± 2.78	73.63–76.21	0.355
p-value		0.024			0.718		

Different superscript letters within each column indicates significant difference (Adjusted *p*-value, Tukey’s HSD)

### Compressive strength results

**Fig. 2:**
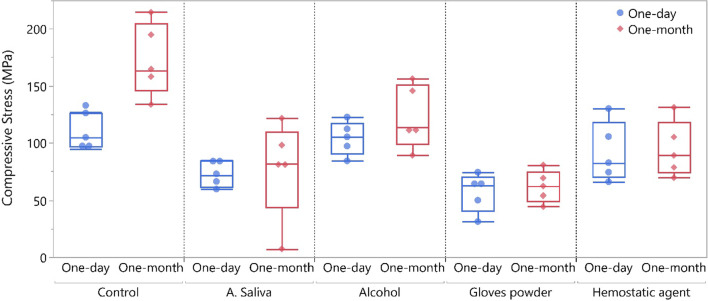
Boxplot showing compressive strength (MPa) values for different tested groups at 1 day and 1 month post-photocuring

The repeated measures ANOVA revealed that different tested groups (*p* < 0.001) and storge time (*p* = 0.018) had a significant effect on the compressive strength (MPa) while the interaction between groups and storage time resulted in an insignificant effect on the compressive strength (MPa) (*p* = 0.090). The compressive strength (MPa) results of tested groups are presented in Table 3 and Fig. 2. At 1 day post-photocuring, control and alcohol groups showed the significantly high compressive strength (MPa) value compared to gloves powder (*p* = 0.01 and 0.028). Artificial saliva and hemostatic agent groups showed an insignificant difference compared to all other groups (*p* > 0.05). At 1-month post-photocuring, control group showed the significantly high compressive strength (MPa) value compared to all groups (*p* < 0.05). Alcohol resulted in significant high compressive strength (MPa) compared to artificial saliva and gloves powder (*p* = 0.043 and 0.003) with insignificant difference between artificial saliva and gloves powder. Hemostatic agent showed insignificant results compared to all other groups except control (*p* < 0.001). One-month post-photocuring resulted in significant increase in the compressive strength for control group only (*p* = 0.001), while for other groups, it didn’t affect he compressive strength significantly (*p* > 0.05).

**Table 3:** Compressive strength (MPa) for tested group at 1-day and at 1-month post-photocuring

	One-day			One-month			*p*-value
	n	Mean ± SD	95%CI	n	Mean ± SD	95%CI	
Control	5	110.42^a^ ± 15.10	91.68–129.17	5	172.87^a^ ± 31.92	133.24–212.50	0.001
A. Saliva	5	72.45^ab^ ± 11.33	58.39–86.52	5	77.71^c^ ± 42.77	24.60–130.82	0.752
Alcohol	5	104.22^a^ ± 14.62	86.07–122.37	5	122.66^b^ ± 27.70	88.26–157.06	0.751
Gloves powder	5	56.71^b^ ± 16.66	36.02–77.39	5	61.97^c^ ± 13.89	11.09–88.07	0.751
Hemostatic agent	5	91.59^ab^ ± 26.09	59.20–123.98	5	94.55^bc^ ± 24.34	64.34–124.77	0.858
p-value		< 0.001			< 0.001		

Different superscript letters within each column indicates significant difference (Adjusted *p*-value, Tukey’s HSD)

## Discussion

Resin composites had enabled minimal invasive dentistry to preserve healthy tooth structure offering an aesthetically pleasing appearance. Resin composites are used as esthetic restorations for anterior and posterior teeth, as pits and fissure sealants, as cavity liners, and as a core build-up material [17]. Bulk filling of cavity would be advantageous if compared with incremental layering of resin composite in reducing treatment time for cavity restoration, polymerization stress, contraction stress and improving esthetic quality [18]. Flowable composite can be used as a stress-breaker intermediate layer between restoration and substrate to relieve the stress associated with polymerization shrinkage [19]. Our study used bulk-fill flowable resin composite as it has become common to be used by the clinicians especially in class II subgingival caries, root caries, deep cavities. These areas are difficult to be properly isolated and are accidentally liable to contamination. The mechanical properties of resin composites are significantly influenced by the filler particle shape, size range, and volume content [20]. The introduction of nanometer sized particles is thought to offer superior esthetics and polishability in addition to excellent wear resistance and strength [20]. Nanohybrid resin composites include a mixture of nanosized and conventional filler particles [20].

The current study used artificial saliva among the used contaminants; because salivary contamination to resin composite during packing is common in some clinics where rubber dam is not used; or when leakage occurs due to improper use of rubber dam. The study also used alcohol among contaminants because many dentists may use alcohol to lubricate the composite instruments to facilitate its handling [21]. Aluminium chloride (AlCl_3_) is one of the hemostatic agents used at a concentration between 0 and 25% to promote hemostasis before placement of a restoration by protein precipitation and constriction of blood vessels [22]. Aluminium chloride (25%) was used in the current study because of its minimum tissue irritation, ease of use and effective results [22]. Many clinicians manipulate the resin composite material during packing with gloves containing powder, causing its contamination. Therefore, powdered gloves were used in this study as contaminant during packing resin composite.

Microhardness is one of the most useful properties to assess because it is closely correlated with resistance to abrasion when used for restoration in load bearing areas [1]. Compressive strength is a vital test for the selection of core material since most of the masticatory forces are of compressive nature [16].

It was observed that the tested contaminants did not affect the resin composite surface microhardness at 1 day and 1 month post-photocuring except of alcohol at 1-day. However, the compressive strength of resin composite was significantly decreased after being contaminated with the tested contaminants at 1-day and 1-month post-photocuring. This might be due to the application of the contaminants between the two increments, not on the surface of the specimen. Vickers hardness test could only assess the surface microhardness of resin composite specimen [23]. Moreover, Celluloid strip and glass slab were used in specimen preparation, so oxygen inhibited layer was not formed on the resin composite specimen surface [23]. Absence of oxygen inhibited layer on the specimen surface might be the cause of proper resin polymerization with subsequent increased surface microhardness.

Our results were inconsistent with Widiandini et al*.* [24] who demonstrated that the salivary contamination did not affect the compressive strength of nanohybrid composite. This inconsistence of results could be due to the application of the contaminant in that study onto the surface of the specimen, while the contaminants in the current study were applied in-between increments. However, Cobanoglu et al. [25] stated that when saliva encounters the dentin surface, a saliva layer is deposited on dentin surface; water is evaporated and leaves a glycoprotein layer. Likewise, Shimazu et al. [26] found that the bond strength among composite resin layers is reduced when it is contaminated with saliva. Similarly, significant decrease of the compressive strength of resin composite contaminated with artificial saliva could be due to presence of organic adherent layer and other elements of saliva in between increments interfering with proper polymerization.

The Food and Drug Administration (FDA) defined ethanol as a liquid that simulates fatty foods and alcoholic beverages. In several studies, ethanol led to degradation or “softening” of composites and reduction of its microhardness [27]. It was previously revealed that ethanol has a more aggressive potential and causes higher water sorption and solubility than water or artificial saliva [28]. Ethanol contamination was found in a previous study [29] to inhibit the resin composite polymerization causing significant decrease of surface microhardness, similar to our study, particularly after 1-day post-photocuring. However, the routine surface polishing can remove the affected outer layer resulting in hardness that was similar to the uncontaminated resin composite [29].

It was previously postulated that manual manipulation of resin composite with powdered latex gloves should be avoided [30]. Martins et al. [12] found that the flexural strength of resin composite manipulated with powdered gloves was reduced. It was previously revealed that sulfides released from latex gloves inhibited the polymerization of the silicone in impression materials based on polyvinyl siloxanes, when it reacts with chloroplatinic acid from silicones [30]. The results of the current study confirmed previous studies. Our findings could be the result of the physical barrier action of powder particles deposited on the resin composite interfering with complete polymerization and subsequently affecting the mechanical properties namely the compressive strength of resin composite.

It was previously demonstrated that the acidic pH of AlCl_3_ used as hemostatic agent resulted in smear layer removal, and dentin etching effects [13, 14]. Similarly, the chloride ions could penetrate the uncured resin matrix or in-between the fillers and might slightly etch the resin matrix or the fillers reducing the compressive strength of the resin composite specimen.

At 1-month post-photocuring, the control group recorded a significantly higher compressive strength mean value when compared to 1-day post-photocuring value. Our findings confirmed the results of Gornig et al. [31]. However, the contaminated groups showed no significant difference between compressive strength mean value at 1-day and 1-month post-photocuring. The stability of compressive strength of contaminated resin composite at 1-month post-photocuring indicates the negative effect of hemostatic agent, alcohol, artificial saliva and powdered gloves on the compressive strength.

The findings of this in vitro study have to be seen in light of some limitations such as small sample size, absence of humidity and pH conditions of the oral cavity. However, the current study raises the importance of the clinician awareness to avoid contamination of resin composite during its packing inside the prepared cavity to enhance the restoration longevity.

## Conclusions

Under the limitations of the current study, it was concluded that contamination of bulk-fill flowable nanohybrid resin composite during packing with either hemostatic agent, alcohol, artificial saliva, or powdered gloves decreased the compressive strength at one-day and 1-month post-photocuring. Whereas contamination of resin composite did not significantly affect its surface microhardness.

## Future scope

Further studies are recommended to evaluate the effect of contamination of bulk-fill resin composite on its color stability, physical and mechanical properties.

## Supplementary Information

**Additional file 1**. Original Data of microhardness and compressive strength.

## Data Availability

The datasets analyzed during the current study are available in the additional Additional file 1 or could be obtained from the corresponding author “nawal.aidaros@acu.edu.eg”.

## Abbreviations

SD: Standard deviation
MPa: Mega pascal
sec: Second
LED: Light emitting diode
HV: Vickers hardness
CS: Compressive strength
ANOVA: Analysis of variance
AlCl_3_: Aluminium chloride
FDA: Food and Drug Administration
pH: Potential of hydrogen
NaCl: Sodium chloride
KCl: Potassium chloride
NaH_2_PO_4_·2H_2_O: Sodium dihydrogen dehydrates
Na_2_S·9H_2_O: Hydrated sodium sulfide
